# Different Dietary Proportions of Fish Oil Regulate Inflammatory Factors but Do Not Change Intestinal Tight Junction ZO-1 Expression in Ethanol-Fed Rats

**DOI:** 10.1155/2017/5801768

**Published:** 2017-12-13

**Authors:** Yi-Wen Chien, Hsiang-Chi Peng, Ya-Ling Chen, Man-Hui Pai, Hsiao-Yun Wang, Hsiao-Li Chuang, Suh-Ching Yang

**Affiliations:** ^1^School of Nutrition and Health Sciences, Taipei Medical University, Taipei 110, Taiwan; ^2^Research Center of Geriatric Nutrition, College of Nutrition, Taipei Medical University, Taipei 110, Taiwan; ^3^Department of Nutrition and Health Sciences, Chang Gung University of Science and Technology, Taoyuan 333, Taiwan; ^4^Department of Anatomy, Taipei Medical University, Taipei 110, Taiwan; ^5^National Applied Research Laboratories, National Laboratory Animal Center, Taipei 115, Taiwan

## Abstract

Sixty male Wistar rats were fed a control or an ethanol-containing diet in groups C or E. The fat compositions were adjusted with 25% or 57% fish oil substituted for olive oil in groups CF25, CF57, EF25, and EF57. Hepatic thiobarbituric acid-reactive substance (TBARS) levels, cytochrome P450 2E1 protein expression, and tumor necrosis factor- (TNF-) *α*, interleukin- (IL-) 1*β*, IL-6, and IL-10 levels, as well as intracellular adhesion molecule (ICAM)-1 levels were significantly elevated, whereas plasma adiponectin level was significantly reduced in group E (*p* < 0.05). Hepatic histopathological scores of fatty change and inflammation, in group E were significantly higher than those of group C (*p* < 0.05). Hepatic TBARS, plasma ICAM-1, and hepatic TNF-*α*, IL-1*β*, and IL-10 levels were significantly lower, and plasma adiponectin levels were significantly higher in groups EF25 and EF57 than those in group E (*p* < 0.05). The immunoreactive area of the intestinal tight junction protein, ZO-1, showed no change between groups C and E. Only group CF57 displayed a significantly higher ZO-1 immunoreactive area compared to group C (*p* = 0.0415). 25% or 57% fish oil substituted for dietary olive oil could prevent ethanol-induced liver damage in rats, but the mechanism might not be related to intestinal tight junction ZO-1 expression.

## 1. Introduction

Excessive or chronic alcohol consumption can lead to liver damage through various pathogenic mechanisms. Three primary types of alcohol-induced liver damage include fatty liver, hepatitis, and cirrhosis [1]. Alcohol-induced liver damage is related to an increased NADH/NAD^+^ ratio which promotes fatty acid synthesis and lipid accumulation in liver cells, oxidative stress caused by increased CYP2E1 activity, and an increased endotoxin level which triggers Kupffer's cell activation and inflammatory processes [2–4]. However, the pathogenic mechanisms are complicated and remain obscure.

There is an emerging theory that chronic ethanol abuse dislocates the tight junction (TJ) structure of the intestinal epithelium, which allows bacterial translocation from the intestines into the in vivo circulation thereby inducing hepatic inflammation [5]. It was indicated that higher endotoxin levels were observed in alcoholic liver disease (ALD) patients, and gut leakage seemed to be the main cause [6, 7]. Endotoxins, also known as lipopolysaccharides (LPSs), are derived from the cell walls of gram-negative bacteria. Animal studies also showed that ALD could be prevented when the intestinal microflora was removed by antibiotics [8–10]. Our previous studies also indicated that epidermal growth factor or synbiotics exhibited hepatoprotective effects through ameliorating the intestinal permeability and microbiota in rats under chronic ethanol feeding [11, 12]. Those previous findings powerfully indicated that intestinal barrier disturbances caused by ethanol abuse are the principal pathway of endotoxemia in ALD.

The consumption level and type of dietary fat can influence the progression of liver injury in ALD. It was indicated that diets rich in saturated fatty acids (SFAs) or medium-chain triglycerides (MCTs) protect against liver injury in rats and mice under chronic ethanol feeding, but diets containing polyunsaturated fatty acids (PUFAs) aggravate liver damage induced by ethanol intake [13–15]. However, there were some limitations of those previous studies. First, only one type of fat was used in each experimental diet. Second, the effects on other organs or tissues were not detected.

Fish oil contains abundant levels of eicosapentaenoic acid (EPA) and docosahexaenoic acid (DHA), which are known as n-3 PUFAs. Based on a majority of studies, fish oil (or n-3 PUFAs) is considered to have beneficial effects, including immune regulation, vascular protection, and lipid metabolism modulation [16–18]. However, few studies have discussed the relationship between fish oil and ALD, particularly those focused on intestinal integrity. According to our earlier study, substituting fish oil for olive oil under ethanol exposure improved the fecal microbiota composition; however, effects on intestinal pathological changes in ethanol-fed rats are still unclear. Thus, we hypothesized that fish oil may have a hepatoprotective effect in ethanol-fed rats by means of maintaining the epithelial barrier function in the intestines and further inhibiting the appearance of endotoxin in the circulation. This animal study was performed to investigate the proposed hypothesis.

## 2. Materials and Methods

### 2.1. Animals

Sixty male Wistar rats (8-weeks old, 160~180 g) provided by BioLASCO Taiwan (Ilan, Taiwan) were acclimatized in individual cages at 22 ± 2°C with 50%~70% humidity and a 12 h light/dark cycle for 1 week with a standard rodent diet (LabDiet 5001 Rodent Diet; PMI Nutrition International, St. Louis, MO, USA). The Institutional Animal Care and Use Committee of Taipei Medical University approved all procedures in this study.

### 2.2. Study Protocol

Rats were divided into groups according to their plasma aspartate transaminase (AST) and alanine transaminase (ALT) activities after 1 week of acclimation in order to ensure there was no significant difference among groups in plasma AST and ALT activities at the beginning of the study. Rats were fed with either a control diet or ethanol diet, in which the fat composition of both diets was adjusted with 25% (7.1 g fish oil/kg diet, 6% of total calories) or 57% (16.2 g fish oil/kg diet, 15% of total calories) fish oil substituted for olive oil. Thus, there were six groups in this study: C (control), CF25 (control with 25% fish oil), CF57 (control with 57% fish oil), E (ethanol), EF25 (ethanol with 25% fish oil), and EF57 (ethanol with 57% fish oil). Rats in groups E, EF25, and EF57 were fed an ethanol-containing liquid diet (35% of calories from ethanol) which was modified from Lieber-DeCarli formula [19], while rats in groups C, CF25, and CF57 were pair-fed with an isoenergetic diet without ethanol by substituting ethanol-derived calories with maltodextrin [16]. One gram of fish oil (VIVA Omega-3^™^) which was provided by Viva Life Science (Costa Mesa, CA, USA) contains 350 mg EPA and 250 mg DHA. Monounsaturated fatty acid (MUFA)/PUFA ratios of the diets without fish oil and with 25% and 57% fish oil substitutions were 0.4, 0.7, and 1.5, respectively [16].

Rats were anesthetized and sacrificed after 8 weeks. Blood samples were collected via the ventral aorta into heparin-containing tubes and centrifuged at 1200 ×g for 15 min (at 4°C); then plasma was collected and stored at −80°C until analysis. Liver tissues were rapidly excised, and a small portion of the liver specimen was cut and fixed in a 10% formaldehyde solution. The remaining liver tissues were stored at −80°C for further analysis. Moreover, jejunum tissue (2 cm of the middle section) of the small intestine was excised and fixed in a 10% formaldehyde solution.

### 2.3. Measurements and Analytical Procedures

#### 2.3.1. Liver Function Indicators

The most commonly used indicators of liver damage are plasma AST and ALT activities which were measured with the ADVIA® 1800 Chemistry System (Siemens Healthcare Diagnostics, Eschborn, Germany) in this study.

#### 2.3.2. Hepatic Histopathological Examination

Liver tissues were fixed in a 10% formaldehyde solution and embedded in paraffin. Paraffin sections were cut and stained with hematoxylin and eosin (H&E) and trichrome stains. Experienced pathologists blinded to the experimental data carried out the semiquantitative histological evaluation of liver specimens according to the degree of tissue damage, which was scored on a scale of 0 = absent, 1 = trace, 2 = mild, 3 = moderate, and 4 = severe.

#### 2.3.3. Hepatic Antioxidative Status

*(1) Plasma and Hepatic Lipid Peroxidation*. One gram of liver tissue was added to 4 mL of buffer containing 0.25 mM phenylmethylsulfonyl fluoride, 0.25 mM sucrose, and 10 mM Tris-HCl (pH 7.4) and then homogenized and centrifuged at 10^4^ ×g for 15 min at 4°C. Supernatants of the liver homogenate and plasma sample were analyzed for lipid peroxidation by measuring the concentration of thiobarbituric acid-reactive substances (TBARSs) as described previously [20].

*(2) CYP2E1 Protein Expression*. The method of microsome preparation from liver tissues was described previously [19]. Sodium dodecyl sulfate polyacrylamide gel electrophoresis (SDS-PAGE, 10%) was used to separate microsomal proteins (30 *μ*g). Proteins were electroblotted onto polyvinylidene difluoride transfer membranes, and the membranes were separately incubated with mouse monoclonal anti-rat CYP2E1 (Oxford Biomedical Research, Oxford, MI, USA) or mouse anti-actin monoclonal antibodies (Chemicon International, Temecula, CA, USA), then samples were treated with goat anti-mouse immunoglobulin G (IgG)-horseradish peroxidase (HRP) (Chemicon International) and detected with a Western Lightning kit (PerkinElmer Lifesciences, Boston, MA, USA). An Image-Pro Plus 4.5 software analysis was used to quantify the bands.

#### 2.3.4. Inflammatory Response

*(1) Cytokine Measurements*. Ice-cold buffer (1.5 mL) containing 50 mM Tris (pH 7.2), 150 mM NaCl, 1% Triton-X, and 0.1% protease inhibitor was added to the liver tissue (0.5 g) and then homogenized and shaken on ice for 90 min. The homogenized solution was centrifuged at 3000 ×g and 4°C for 15 min. A DuoSet® rat TNF-*α* kit, a rat IL-1*β*/IL-1F2 kit, a rat IL-6 kit, and a rat IL-10 kit (R&D Systems, Minneapolis, MN, USA) were used to analyze the supernatant according to assay kit instructions. A microplate reader (Molecular Devices, Sunnyvale, CA, USA) was used to read the optical density (OD) at 450 nm for all cytokines.

*(2) Plasma Adiponectin Concentration*. An enzyme-linked immunosorbent assay (ELISA) kit (AssayMax Rat Adiponectin ELISA kit Assaypro, St. Charles, MO, USA) was used to measure the plasma adiponectin concentration. The OD was the same as for the cytokine measurements.

*(3) Cell Adhesion Molecule Measurement*. Plasma VCAM-1 and ICAM-1 levels were, respectively, determined with a rat ICAM-1/CD54 Quantikine ELISA kit (R&D Systems, Minneapolis, MN, USA) and Cell Adhesion Molecule 1 Assay Kit (USCN Life Science, Wuhan, China). Procedures followed the manufacturer's instructions. The OD was the same as for the cytokine measurements.

#### 2.3.5. Small-Intestinal Histopathological Examination

*(1) H&E Dye Staining*. Jejunum tissue (2 cm of the middle section) was fixed in 10% formaldehyde and embedded in paraffin. Paraffin sections were cut and stained with H&E dye. A semiquantitative histological evaluation was carried out by a trained pathologist who was blinded to the treatment groups and visually evaluated the degree of tissue injury, according to Chiu's Score Classification of Small-Intestinal Injury [21]. The grading ranges 0~5, the same as described by Yuan et al. [22].

*(2) TJ Protein ZO-1 Immunohistochemical Staining*. The method of ZO-1 immunohistochemical (IHC) staining was described previously [23]. Tissue sections were deparaffinized and incubated with a primary antibody against ZO-1 (1 : 300, Abcam, Cambridge, UK) overnight at 4°C, followed by incubation with a biotinylated secondary antibody (1 : 300, Nippon Chemi-Con, Tokyo, Japan) for 1 h at room temperature. After carrying out the reaction with the peroxidase-linked avidin-biotin complex (Vector) for 1 h at room temperature, a diaminobenzidine solution kit (Vector) was used to detect ZO-1 immunoreactivity. The “count/size” and “area” commands were used to determine the intensity of ZO-1 immunoreactivity.

*(3) Plasma Endotoxin Levels*. Plasma endotoxin levels were measured using a Limulus Amebocyte Lysate Kit (Associates of Cape Cod, East Falmouth, MA, USA). A microplate reader (Molecular Devices) was used to read the OD at 405 nm.

### 2.4. Statistical Analysis

Data are presented as the mean ± standard error of the mean (SEM). SAS software vers. 9.4 (SAS Institute, Cary, NC, USA) and Student's *t*-tests were used to determine statistical differences between groups C and E. A one-way analysis of variance (ANOVA) followed by Duncan's new multiple range test was used to determine statistical differences among groups C, CF25, and CF57 and groups E, EF25, and EF57. A two-way ANOVA was used to confirm the interaction between ethanol and fish oil. *p* values of <0.05 were regarded as statistically significant.

## 3. Results

### 3.1. Food Intake and Ethanol Consumption

No difference was found in food intake among the six groups (group C: 74.8 ± 3.9 kcal/day; group CF25: 74.1 ± 3.9 kcal/day; group CF57: 74.3 ± 4.1 kcal/day; group E: 76.4 ± 3.2 kcal/day; group EF25: 72.0 ± 3.2 kcal/day; and group EF57: 70.1 ± 3.0 kcal/day). The average ethanol consumption in groups E, EF25, and EF57 was 11.4 ± 0.2, 11.3 ± 0.2, and 11.1 ± 0.2 g/kg BW/day, respectively. There was no difference among these ethanol-intake groups.

### 3.2. Body Weight and Relative Liver Weight

Final body weights are shown in Table 1. There was no difference in final body weights between groups C and E. However, final body weights in groups EF25 and EF57 were significantly lower than that of group E (*p* < 0.05). The relative liver weight in group E was significantly higher compared to that of group C (*p* < 0.05). However, the relative liver weights exhibited no differences among groups E, EF25, and EF57.

### 3.3. Hepatic Histopathological Examination

After 8 weeks of feeding, plasma AST and ALT activities of group E were significantly higher than those of group C (*p* < 0.05, Table 2). However, plasma AST activities in groups EF25 and EF57 were significantly lower compared to those of group E (*p* < 0.05).

**Figure 1:**
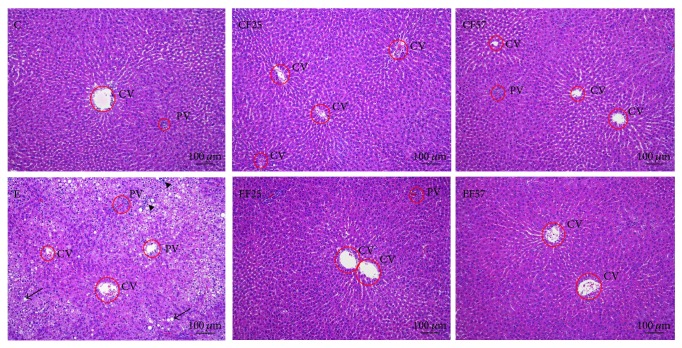
Effects of fish oil on H&E staining of liver tissue sections in rats with chronic ethanol feeding. CV: central vein; PV: portal vein; C: control group; CF25: control diet with fish oil substituted for 25% of olive oil; CF57: control diet with fish oil substituted for 57% of olive oil; E: ethanol group; EF25: alcohol-containing diet with fish oil substituted for 25% of olive oil; EF57: alcohol-containing diet with fish oil substituted for 57% of olive oil. H&E staining showed hepatocyte degeneration and necrosis accompanied by inflammatory cell infiltration (triangle) in group E. Moreover, fatty changes (arrow) were also found in group E.

Histopathological scores of the livers are presented in Table 3. Fatty changes (including macrovesicular and microvesicular), inflammatory cell infiltration, and cell degeneration and necrosis were observed in group E; however, fatty changes, inflammation, and cell degeneration and necrosis were significantly lower in groups FE25 and FE57 than those in group E (*p* < 0.05). According to Figure 1, H&E staining showed hepatocyte degeneration and necrosis accompanied by fat accumulation and inflammatory cell infiltration.

### 3.4. Oxidative Stress

**Figure 2:**
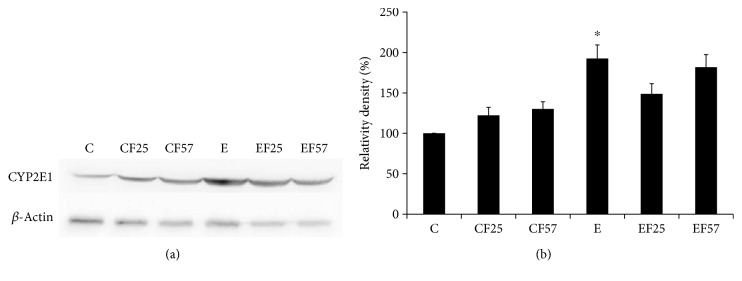
Hepatic CYP2E1 protein expressions in each group. Values are expressed as the mean ± SEM. C: control group; CF25: control diet with fish oil substituted for 25% of olive oil; CF57: control diet with fish oil substituted for 57% of olive oil; E: ethanol group; EF25: alcohol-containing diet with fish oil substituted for 25% of olive oil; EF57: alcohol-containing diet with fish oil substituted for 57% of olive oil. Bars with ∗ significantly differ between groups C and E at the *p* < 0.05 level according to Student's *t*-tests.

TBARS concentrations and CYP2E1 expressions are considered indicators for evaluating the hepatic antioxidative status. Results of plasma and hepatic TBARS concentrations are given in Table 4. Plasma and hepatic TBARS concentrations were significantly higher in group E (*p* < 0.05); however, both plasma and hepatic TBARS concentrations were significantly lower in groups EF25 and EF57 than those in group E (*p* < 0.05). As shown in Figure 2, CYP2E1 expression in group E was significantly higher than that in group C (*p* < 0.05); however, there were no differences among groups E, EF25, and EF57.

### 3.5. Inflammatory Responses

Rats in group E showed significantly elevated TNF-*α*, IL-1*β*, IL-6, and IL-10 concentrations compared to rats in group C (*p* < 0.05, Table 5). Hepatic TNF-*α*, IL-1*β*, IL-6, and IL-10 levels were significantly lower in groups EF25 and EF57 than those in group E (*p* < 0.05).

In addition, group E showed the significantly lowest plasma adiponectin concentration among all groups (*p* < 0.05, Table 6). Further, plasma adiponectin levels were significantly higher in groups EF25 and EF57 than that in group E (*p* < 0.05).

Plasma VCAM-1 and ICAM-1 levels in each group are shown in Table 7. Plasma VCAM-1 and ICAM-1 levels of group E were significantly higher than those of group C (*p* < 0.05). However, plasma VCAM-1 concentrations were significantly lower in groups EF25 and EF57 compared to group E (*p* < 0.05). The plasma ICAM-1 concentration in group EF25 was significantly lower than that in group E (*p* < 0.05).

### 3.6. Small-Intestinal Histopathological Examination and the TJ Protein ZO-1 Distribution

**Figure 3:**
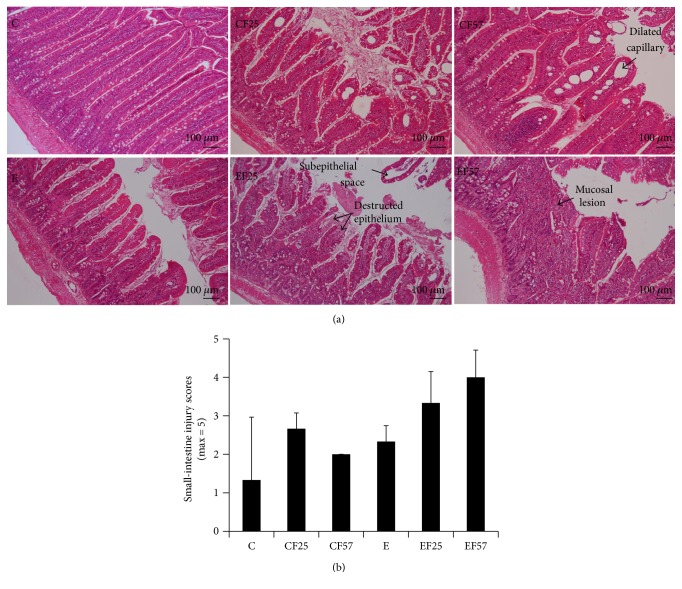
Score of small-intestinal injury in each group. C: control group; CF25: control diet with fish oil substituted for 25% of olive oil; CF57: control diet with fish oil substituted for 57% of olive oil; E: ethanol group; EF25: alcohol-containing diet with fish oil substituted for 25% of olive oil; EF57: alcohol-containing diet with fish oil substituted for 57% of olive oil. (a) Representative histological images of rats in all groups at 100x magnification. (b) Quantification of the small-intestinal injury score among groups.

**Figure 4:**
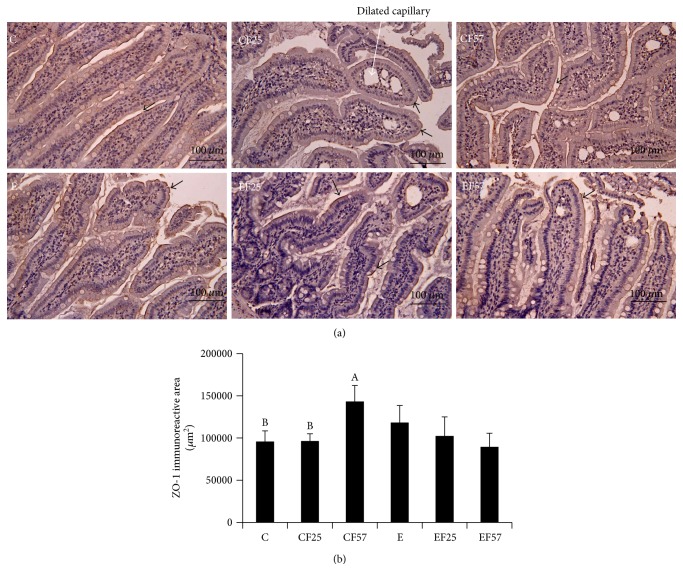
IHC staining of the tight junction protein, ZO-1, in the small-intestinal mucosa in each group. C: control group; CF25: control diet with fish oil substituted for 25% of olive oil; CF57: control diet with fish oil substituted for 57% of olive oil; E: ethanol group; EF25: alcohol-containing diet with fish oil substituted for 25% of olive oil; EF57: alcohol-containing diet with fish oil substituted for 57% of olive oil. (a) Representative histological images of rats in all groups at 200x magnification. Arrows indicate ZO-1-positive areas. The normal small intestine exhibited intact epithelium with marked dark-brown ZO-1 expression. (b) Quantification of ZO-1-immunoreactive areas among groups. Bars with different letters (A, B) significantly differ among groups C, CF25, and CF57 at the *p* < 0.05 level according to a one-way analysis of variance (ANOVA) followed by Duncan's new multiple range test.

According to the Chiu's Score Classification of Small-Intestinal Injury, scores of small-intestinal injury are shown in Figure 3(b). There were no differences among all groups, but groups E, EF25, and EF57 showed a higher trend compared to group C. Scores ranged 2~4, which means the presence of cellular lysis, increased spacing among villosities, structural destruction of the villosities, and so forth (Figure 3(a)). ZO-1 expression in the small-intestinal mucosa was examined by IHC, which revealed that the epithelial structure differed among groups (Figure 4). In group C, the epithelium of the small-intestinal mucosa was intact. Compared to groups C and CF25, group CF57 displayed a significantly larger ZO-1 immunoreactive area (*p* < 0.05). However, there was no change between groups C and E and even among the ethanol-intake groups.

### 3.7. Plasma Endotoxin Level

As shown in Table 8, the plasma endotoxin level was significantly higher in group E compared to that of group C (*p* < 0.05). However, groups EF25 and EF57 presented significantly lower plasma endotoxin concentrations compared to group E (*p* < 0.05).

## 4. Discussion

Similar to our previous studies, the average ethanol intake was 11.1~11.4 g/kg BW/day in the ethanol-intake groups, which would be comparable to heavy drinkers in humans (more than 50~60 g/day of absolute alcohol) after conversion of animal doses to a human equivalent based on body surface areas [16, 17].

Rats fed with the ethanol-containing liquid diet (group E) for 8 weeks showed a slight loss of body weight. However, when rats simultaneously consumed ethanol and fish oil (groups EF25 and EF57), the final body weights significantly decreased (Table 1). Fish oil is associated with a body weight-loss effect in high-fat diet-induced obese animal studies [24, 25]. The potential antibody fat mechanisms of fish oil were suggested to include increased plasma adiponectin levels [25], increased adipocyte apoptosis [26], and altered fat oxidation [27]. Therefore, effects of ethanol and fish oil on adipose tissues should be checked in future studies.

Higher AST and ALT activities, hepatic lipid accumulation, and inflammatory cell infiltration were observed in group E rats (Tables 2 and 3). Ethanol abuse induces hepatic fatty liver and inflammation as proven by hundreds of studies [28] and also by our previous studies [11, 12, 20]. Ethanol-induced pathological alterations in the liver are caused by abnormal lipid metabolism, an imbalance between pro- and anti-inflammatory cytokines, and an elevated plasma endotoxin level [20]. In the present study, fish oil displayed hepatoprotective effects in rats fed with ethanol-containing liquid diets based on the lower ALT activity and hepatic histopathological scores (Tables 2 and 3). We speculated that the protective mechanisms of fish oil in rats with ethanol-induced liver injuries might be associated with antilipid accumulation, antioxidative stress (Table 4), and immunoregulatory effects (Table 5). The antioxidative potential of fish oil is controversial. Ramaiyan et al. suggested that fish oil which was added to the AIN-70 diet (50 g/kg diet, 2.5 g/kg body weight) decreased hepatic TBARS contents in rats [29]. On the contrary, Tsuduki et al. indicated that the consumption of a fish oil diet (fish oil: safflower oil ratio of 50 : 50 g/kg of diet, 5.53 g/kg body weight) for 28 weeks significantly increased plasma and hepatic TBARS contents in male SAMP8 mice [30]. In the present study, fish oil intake levels were 1.07 and 2.43 g/kg body weight in rats fed with fish oil, which were similar to levels in Kikugawa et al.'s study [27]. Therefore, appropriate proportions of SFAs, MUFAs, and PUFAs are very important for preventing diseases induced by oxidative stress [31]. On the other hand, several studies substantiated that the anti-inflammatory effects of fish oil were related to the production of E-series resolvins (from EPA) and D-series resolvins (from DHA) through the cyclooxygenase (COX)-2 pathway [32]. In our previous study, we also found that fish oil normalized hepatic pro- and anti-inflammatory cytokine secretions in rats under chronic ethanol abuse [20].

Adiponectin inhibits expressions of ICAM-1 and VCAM-1 through inhibiting nuclear factor (NF)-*κ*B activation and has several antiatherogenic and anti-inflammatory properties [33]. Moreover, several animal models indicated that hypoadiponectinemia and altered hepatic adiponectin signaling induced by chronic ethanol intake are associated with steatosis and inflammation [34]. We also found that the plasma adiponectin level significantly decreased; in contrast, plasma ICAM and VCAM levels increased in rats fed with ethanol only (group E in Tables 6 and 7). However, when ethanol-fed rats ingested fish oil, lower plasma adiponectin levels were ameliorated; in addition, plasma ICAM and VCAM levels were reduced (groups EF25 and EF57 in Tables 6 and 7). Dietary intake of omega-3 (n-3) PUFAs has emerged as an important way to modify cardiovascular risk by regulating the endothelial expression of adhesion molecules and adipokines, such as ICAM, VCAM, and adiponectin in cardiovascular diseases and diabetes [35, 36]. To our best knowledge, this is the first study to find that fish oil substitution in the diet can increase plasma adiponectin levels and decrease plasma adhesion molecules in rats under chronic ethanol feeding. Further studies are necessary to clarify the relationship between fish oil and lipid metabolism-related molecular factors of the adiponectin regulatory pathway.

A previous study indicated that ethanol and its metabolites (such as acetaldehyde) destroy intestinal epithelial TJ proteins, including ZO-1 and occludin, and thus cause poor integrity of the gut barrier in a chronic ethanol-intake animal model [37]. In this study, no differences in small-intestinal injury or the ZO-1 immunoreactive area were found in rats fed with ethanol (group E in Figures 3 and 4); nevertheless, rats that were fed with ethanol chronically for 8 weeks (group E) showed significantly higher plasma endotoxin levels (Table 8). Thus, data on intestinal histopathology in this study were insufficient to explain the hyperendotoxinemia in rats exposed to chronic ethanol intake. The other TJ protein, occludin, or the intestinal permeability regulator, zonulin, should be measured in future studies [38]. Interestingly, when fish oil was substituted for olive oil in the nonethanol-containing diet (group CF57), a significantly larger ZO-1 immunoreactive area was detected (Figure 4). In contrast, no obvious change in the ZO-1 immunoreactive area was observed in rats fed with fish oil and an ethanol-containing diet (group EF57, Figure 4). The feeding pattern which mixed fish oil into the ethanol-containing liquid diet might be a possible reason for the weakened protective effect on the intestinal epithelium by fish oil supplements. However, we still found that fish oil ameliorated high plasma endotoxin levels in rats under chronic ethanol-intake (groups EF25 and EF57, Table 8). Mani et al. indicated that postprandial serum endotoxin concentrations increased after a meal rich in SFAs and decreased with higher n-3 PUFA intake in a pig model [39]. A previous study also demonstrated that the signaling and transport processes for endotoxin are initiated in specialized membrane microdomains called lipid rafts, and oil rich in n-3 PUFAs may unsettle lipid rafts that inhibit greater endotoxin transport [39, 40]. Thus, we propose that the mechanism of n-3 PUFA-enriched fish oil inhibiting endotoxin transport across the intestinal epithelium may be associated with fatty acid regulation of intestinal membrane lipid rafts rather than the structural integrity.

In this study, no dose-response effect of fish oil substitution levels on alcohol-induced liver damage was observed in the hepatic histopathological score or inflammatory factors, including cytokines, adhesion molecules, and adipokines. Therefore, based on our data, taking more fish oil supplements cannot provide greater protective effects against alcoholic liver injuries in rats.

## 5. Conclusions

In conclusion, chronic ethanol feeding elevated the plasma endotoxin level that may trigger inflammatory responses and consequently contribute to liver injury. Moreover, fish oil substituted for olive oil under ethanol exposure inhibited the appearance of endotoxin in the circulation, thus decreasing inflammatory responses which exert a hepatoprotective potential in rats under chronic ethanol feeding. However, the mechanism of decreased plasma endotoxin levels by fish oil supplementation might not be related to improved intestinal structural integrity.

## Figures and Tables

**Table 1:** Final body weights and relative liver weights in each group^1,2,3^.

		—	F25	F57	Ethanol^∗^ and fish oil
Final weight (g)	C	409.5 ± 5.4	410.5 ± 3.6	413.6 ± 6.3	0.0338
	E	397.1 ± 4.4^e^	374.5 ± 5.5^f^	368.0 ± 10.4^f^	
Liver weight (g)	C	10.1 ± 0.2^b^	10.8 ± 0.4^ab^	11.7 ± 0.3^a^	0.3006
	E	12.1 ± 0.3^∗^	12.3 ± 0.8	12.3 ± 0.5	
Relative liver weight (%)	C	2.5 ± 0.0^c^	2.6 ± 0.1^b^	2.8 ± 0.0^a^	0.8601
	E	3.0 ± 0.1^∗^	3.3 ± 0.2	3.3 ± 0.1	

^1^Values are expressed as the mean ± SEM. Means between groups C and E with ∗ significantly differ (*p* < 0.05). Means among groups C, CF25, and CF57 with different superscript letters (a, b, c) significantly differ (*p* < 0.05). Means among groups E, EF25, and EF57 with different superscript letters (e, f) significantly differ (*p* < 0.05). ^2^Relative liver weight: (liver weight/body weight) × 100%. ^3^C: control group; CF25: control diet with fish oil substituted for 25% of olive oil; CF57: control diet with fish oil substituted for 57% of olive oil; E: ethanol group; EF25: alcohol-containing diet with fish oil substituted for 25% of olive oil; EF57: alcohol-containing diet with fish oil substituted for 57% of olive oil.

**Table 2:** Final plasma aspartate transaminase (AST) and alanine transaminase (ALT) activities in each group^1,2^.

(U/L)		—	F25	F57	Ethanol^∗^ and fish oil
ALT	C	48.4 ± 4.4	45.5 ± 2.4	49.1 ± 2.3	0.5782
	E	87.9 ± 12.5^∗^	75.9 ± 5.7	73.6 ± 4.8	
AST	C	83.4 ± 2.5	84.9 ± 3.0	91.5 ± 2.5	0.3965
	E	185.3 ± 18.6^∗^^e^	131.0 ± 13.5^f^	156.0 ± 17.4^f^	

^1^Values are expressed as the mean ± SEM. Means between groups C and E with ∗ significantly differ (*p* < 0.05). Means among groups E, EF25, and EF57 with different superscript letters (e, f) significantly differ (*p* < 0.05). ^2^Details are the same as those described in the footnotes of Table 1.

**Table 3:** Hepatic histopathology scores in each group^1,2^.

		—	F25	F57	Ethanol^∗^ and fish oil
Fatty change (macrovesicular)	C	1.6 ± 0.2	1.2 ± 0.2	1.2 ± 0.2	0.1779
	E	2.8 ± 0.2^∗^^e^	1.8 ± 0.2^f^	1.6 ± 0.2^f^	
Fatty change (microvesicular)	C	0.0 ± 0.0	0.0 ± 0.0	0.0 ± 0.0	<0.0001
	E	1.8 ± 0.2^∗^^e^	0.0 ± 0.0^f^	0.0 ± 0.0^f^	
Inflammatory cell infiltration	C	1.6 ± 0.2^a^	1.8 ± 0.2^a^	0.6 ± 0.2^b^	0.0635
	E	2.8 ± 0.2^∗^^e^	1.8 ± 0.2^f^	1.6 ± 0.4^f^	
Cell degeneration and necrosis	C	1.4 ± 0.2^a^	1.0 ± 0.0^ab^	0.8 ± 0.2^b^	0.4831
	E	3.0 ± 0.0^∗^^e^	2.4 ± 0.2^f^	2.0 ± 0.0^f^	
Bile duct hyperplasia	C	1.4 ± 0.2	1.4 ± 0.2	1.0 ± 0.3	0.1288
	E	1.0 ± 0.0	1.4 ± 0.2	1.6 ± 0.2	
Fibrosis	C	0.8 ± 0.4	1.4 ± 0.2	0.8 ± 0.2	0.7725
	E	1.0 ± 0.3	1.4 ± 0.2	0.6 ± 0.2	

^1^Values are expressed as the mean ± SEM. Means between groups C and E with ∗ significantly differ (*p* < 0.05). Means among groups C, CF25, and CF57 with different superscript letters (a, b) significantly differ (*p* < 0.05). Means among groups E, EF25, and EF57 with different superscript letters (e, f) significantly differ (*p* < 0.05). ^2^Details are the same as those described in the footnotes of Table 1.

**Table 4:** Thiobarbituric acid-reactive substance (TBARS) concentrations in each group^1,2^.

		—	F25	F57	Ethanol^∗^ and fish oil
Plasma TBARS	C	15.4 ± 0.6	16.5 ± 0.8	15.1 ± 0.5	0.0009
(*μ*M)	E	20.4 ± 0.5^∗^^e^	16.3 ± 0.7^f^	16.0 ± 0.8^f^	
Hepatic TBARS	C	615.4 ± 17.5^a^	532.3 ± 17.8^b^	463.0 ± 25.0^c^	0.328
(nmol/g liver)	E	804.3 ± 29.1^∗^^e^	637.7 ± 26.2^f^	594.1 ± 7.4^f^	

^1^Values are expressed as the mean ± SEM. Means between groups C and E with ∗ significantly differ (*p* < 0.05). Means among groups C, CF25, and CF57 with different superscript letters (a, b, c) significantly differ (*p* < 0.05). Means among groups E, EF25, and EF57 with different superscript letters (e, f) significantly differ (*p* < 0.05). ^2^Details are the same as those described in the footnotes of Table 1.

**Table 5:** Hepatic tumor necrosis factor- (TNF-) *α*, interleukin- (IL-) 1*β*, IL-6, and IL-10 levels in each group^1,2^.

(pg/mg liver)		—	F25	F57	Ethanol^∗^ and fish oil
TNF-*α*	C	62.2 ± 5.5	69.7 ± 3.6	62.7 ± 3.8	0.0118
	E	86.1 ± 4.5^∗^^e^	65.3 ± 5.4^f^	67.8 ± 4.3^f^	
IL-1*β*	C	60.4 ± 3.0^ab^	66.9 ± 2.2^a^	58.6 ± 1.3^b^	0.0012
	E	76.5 ± 1.9^∗^^e^	61.3 ± 2.4^f^	64.6 ± 4.5^f^	
IL-6	C	95.0 ± 4.5	94.1 ± 3.2	87.5 ± 4.9	0.0974
	E	120.6 ± 6.2^∗^^e^	97.2 ± 5.8^f^	100.0 ± 5.3^f^	
IL-10	C	88.2 ± 3.2	89.5 ± 4.9	75.9 ± 5.6	0.0263
	E	115.8 ± 4.4^∗^^e^	92.4 ± 4.4^f^	92.8 ± 3.3^f^	

^1^Values are expressed as the mean ± SEM. Means between groups C and E with ∗ significantly differ (*p* < 0.05). Means among groups C, CF25, and CF57 with different superscript letters (a, b) significantly differ (*p* < 0.05). Means among groups E, EF25, and EF57 with different superscript letters (e, f) significantly differ (*p* < 0.05). ^2^Details are the same as those described in the footnotes of Table 1.

**Table 6:** Plasma adiponectin levels in each group^1,2^.

		—	F25	F57	Ethanol^∗^ and fish oil
Adiponectin	C	15.0 ± 0.5	16.0 ± 0.5	16.5 ± 0.8	0.1805
	E	8.2 ± 0.7^∗^^f^	12.5 ± 1.2^e^	11.2 ± 1.1^ef^	

^1^Values are expressed as the mean ± SEM. Means between groups C and E with ∗ significantly differ (*p* < 0.05). Means among groups C, CF25, and CF57 with different superscript letters significantly differ (*p* < 0.05). Means among groups E, EF25, and EF57 with different superscript letters (e, f) significantly differ (*p* < 0.05). ^2^Details are the same as those described in the footnotes of Table 1.

**Table 7:** Plasma vascular cell adhesion molecule (VCAM)-1 and intercellular adhesion molecular (ICAM)-1 levels of rats in each group^1,2^.

(ng/mL)		—	F25	F57	Ethanol^∗^ and fish oil
VCAM-1	C	124.21 ± 12.16	147.56 ± 16.23	132.45 ± 16.57	0.0085
	E	187.81 ± 33.07^e^	86.13 ± 9.35^f^	83.95 ± 5.29^f^	
ICAM-1	C	28.64 ± 1.24^a^	23.86 ± 1.23^b^	26.02 ± 0.72^ab^	0.1612
	E	36.58 ± 1.27^∗^^e^	26.56 ± 0.79^f^	32.9 ± 1.79^e^	

^1^Values are expressed as the mean ± SEM. Means between groups C and E with ∗ significantly differ (*p* < 0.05). Means among groups C, CF25, and CF57 with different superscript letters (a, b) significantly differ (*p* < 0.05). Means among groups E, EF25, and EF57 with different superscript letters (e, f) significantly differ (*p* < 0.05). ^2^Details are the same as those described in the footnotes of Table 1.

**Table 8:** Plasma endotoxin levels in each group^1,2^.

(EU/ml)		—	F25	F57	Ethanol^∗^ and fish oil
Endotoxin	C	20.71 ± 0.27	19.36 ± 0.82	18.95 ± 0.84	0.0064
	E	24.67 ± 1.22^∗^^e^	17.8 ± 1.87^f^	16.18 ± 1.12^f^	

^1^Values are expressed as the mean ± SEM. Means between groups C and E with ∗ significantly differ (*p* < 0.05). Means among groups E, EF25, and EF57 with different superscript letters (e, f) significantly differ (*p* < 0.05). ^2^Details are the same as those described in the footnotes of Table 1.
